# L1cam promotes tumor progression and metastasis and is an independent unfavorable prognostic factor in gastric cancer

**DOI:** 10.1186/1756-8722-6-43

**Published:** 2013-06-27

**Authors:** Dong-liang Chen, Zhao-lei Zeng, Jing Yang, Chao Ren, De-shen Wang, Wen-jing Wu, Rui-hua Xu

**Affiliations:** 1 State Key Laboratory of Oncology in South China, Sun Yat-sen University Cancer Center, 651 Dong Feng East Load, Guangzhou 510060, China; 2 Department of Medical Oncology, Sun Yat-sen University Cancer Center, Guangzhou, China; 3 Department of Experimental Research, Sun Yat-sen University Cancer Center, Guangzhou, China

**Keywords:** L1cam, Metastasis, PI3K/Akt, Prognosis, Gastric cancer

## Abstract

**Background:**

Previous reports have demonstrated that L1cam is aberrantly expressed in various tumors. The potential role of L1cam in the progression and metastasis of gastric cancer is still not clear and needs exploring.

**Methods:**

Expression of L1cam was evaluated in gastric cancer tissues and cell lines by immunohistochemistry and Western blot. The relationship between L1cam expression and clinicopathological characteristics was analyzed. The effects of L1cam on cell proliferation, migration and invasion were investigated in gastric cancer cell lines both *in vitro* and *in vivo*. The impact of L1cam on PI3K/Akt pathway was also evaluated.

**Results:**

L1cam was overexpressed in gastric cancer tissues and cell lines. L1cam expression was correlated with aggressive tumor phenotype and poor overall survival in gastric cancer patients. Ectopic expression of L1cam in gastric cell lines significantly promoted cell proliferation, migration and invasion whereas knockdown of L1cam inhibited cell proliferation, migration and invasion *in vitro* as well as tumorigenesis and metastasis *in vivo*. The low level of phosphorylated Akt in HGC27 cells was up-regulated after ectopic expression of L1cam, whereas the high level of phosphorylated Akt in SGC7901 cells was suppressed by knockdown of L1cam. Moreover, the migration and invasion promoted by L1cam overexpression in gastric cancer cells could be abolished by either application of LY294002 (a phosphoinositide-3-kinase inhibitor) or knockdown of endogenous Akt by small interfering RNA.

**Conclusions:**

Our study demonstrated that L1cam, overexpressed in gastric cancer and associated with poor prognosis, plays an important role in the progression and metastasis of gastric cancer.

## Background

Gastric cancer is the fourth most common malignancy and second leading cause of cancer-related mortality worldwide [1]. Although it is curable if detected early, most patients are diagnosed at advanced stage and have poor prognosis [2]. Tumor invasion and metastasis are critical steps in determining aggressive tumor phenotype and also constitute the main causes of cancer-related deaths [3]. Because traditional methods do not allow precise prediction of tumor progression and metastasis for patients after surgical resection of the primary tumor, there is an urgent need to identify new molecules that associated with gastric cancer progression and metastasis [4].

L1cam is a 220 kDa multidomain type 1 membrane glycoprotein that belongs to the neuronal immunoglobulin superfamily of cell surface molecules [5]. L1cam contains six IgG-like and five fibronectin-type III domains in the extracellular region, a transmembrane region and a short intracellular cytoplasmic tail [6,7]. L1cam was first described as a neural cell adhesion molecule and has been shown to play an important role in cerebellar cell motility and development of the nervous system as well as neural growth and regeneration [8-10]. Besides neural cells, L1cam is found to be normally expressed in other cell types such as kidney tubule epithelial cells, intestinal crypt cells and myelomonocytic cells [11-13]. Recent reports found L1cam is also expressed in various tumor cells, including colorectal cancer, renal cell carcinoma, ovarian cancer, anaplastic thyroid carcinoma, malignant glioma, recurrent neuroblastoma and cutaneous malignant melanoma, and its expression is associated with tumor progression and invasion [14-20]. Studies have demonstrated that L1cam is able to stimulate many cellular activities via homophilic biding to the extracellular domains of the cells and heterophilic biding to other cell adhesion proteins, integrins, extracellular matrix molecules and cell surface receptors [21-23]. Ectopic expression of L1cam could promote tumor cell proliferation, migration and invasion in several types of cancer, including colon cancer, intrahepatic cholangiocarcinoma, and gallbladder carcinoma [24-26]. In gastric cancer, Kodera et al. reported L1cam was associated with prognosis of pT3-stage patients [27]. However, the biological role and underlying molecular mechanism of L1cam in gastric cancer progression and metastasis is still not known.

Akt (also known as Protein Kinase B) is a serine/threonine-specific protein kinase, which functions as a hub gene to integrate with different cellular signaling pathways [28]. Threonine 308 and serine 473 (two specific amino acid residues of Akt) can be phosphorylated upon full activation of Akt; Akt signaling has been shown to regulate multiple cellular activities, including cell cycle, cell growth, cell proliferation, cell migration/invasion and cell metabolism [29,30]. Activation of Akt signaling pathway has been found to be involved in tumor growth and invasion of some malignant disease [15,25]. However, it is still unknown whether L1cam can activate Akt and promote tumor growth and metastasis in gastric cancer.

In this study, we found L1cam was overexpressed in gastric cancer tissues and cell lines. Expression of L1cam was associated with clinicopathological characteristics and prognosis in gastric cancer patients. Knockdown of L1cam in gastric cancer cell lines significantly reduced cell proliferation, migration and invasion *in vitro* and suppressed tumorigenesis and metastasis in an experimental nude mouse model. Conversely, ectopic expression of L1cam in gastric cells significantly promoted these activities. Moreover, we found that the PI3K/Akt pathway was involved in the L1cam promoted cell proliferation, migration and invasion. These results suggest L1cam plays an important role in the progression and metastasis of gastric cancer and could be used as a new therapeutic target.

## Results

### L1cam is overexpressed in gastric cancer cell lines and tissues

**Figure 1:**
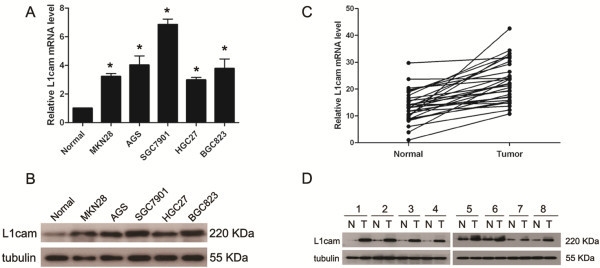
**L1cam is overexpressed in gastric cancer cell lines and primary tumor tissues. (A)** L1cam mRNA expression levels in gastric cancer cell lines compared with normal gastric cancer tissue, (*P < 0.005). **(B)** L1cam protein expression in gastric cancer cell lines and normal gastric cancer tissue. **(C)** L1cam mRNA expression levels in 30 paired gastric cancer tissues and adjacent non-cancerous tissues. **(D)** L1cam protein expression level in paired gastric cancer tissues and adjacent non-cancerous tissues (the representative ones are shown).

L1cam mRNA expression was higher in all five gastric cancer cell lines compared with normal gastric cancer mucosa (Figure 1A). Western blot analysis confirmed overexpression of L1cam in all gastric cancer cell lines (Figure 1B). In matched primary gastric cancer tissues and adjacent normal tissues, the expression of L1cam mRNA was up-regulated by more than 1.5 fold in 19 of 30 (63%) cancer tissues than that of normal tissues (Figure 1C). Western blot showed overexpression of L1cam in 23 of 30 (76%) cancer tissues compared with adjacent normal tissues (Figure 1D).

### Overexpression of L1cam is associated with poor prognosis in gastric cancer

**Figure 2:**
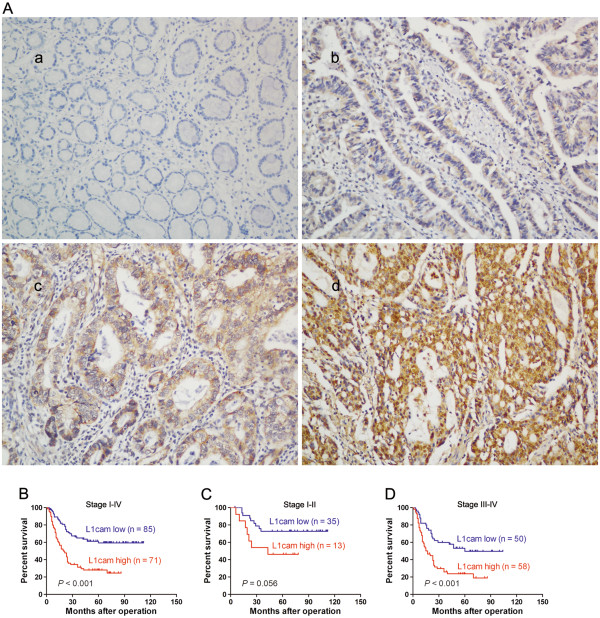
**The prognostic significance of L1cam in gastric cancer patients. (A)** Representative photos of L1cam expression in 156 gastric cancer patients, a, negative staining of L1cam in adjacent normal tissues; b, weak staining of L1cam in well differentiated gastric cancer tissues; c, moderate staining of L1cam in cancer tissues; d, strong staining of L1cam in cancer tissues, amplification (×100). **(B)** Kaplan-Meier analysis of overall survival based on L1cam expression in all 156 patients. **(C)** and **(D)** Kaplan-Meier analysis of overall survival based on L1cam expression in stage I-II **(C)** and stage III-IV gastric cancer patients **(D)**.

To evaluate the clinicopathological significance of L1cam in gastric cancer, immunohistochemistry analysis was performed in 156 gastric cancer samples. As shown in Figure 2A, L1cam protein was mainly located in the cytoplasm and cell membrane of tumor cells. Positive staining was observed in 114 of 156 (73%) cases. The patients were divided into the L1cam low expression group (n = 85) and the L1cam high expression group (n = 71) based on IHC scores. The correlation between L1cam expression and clinicopathological characteristics was listed in Table 1. High expression of L1cam was positively associated with large tumor size (*P* = 0.001), lymph node invasion (*P* = 0.007), peritoneal dissemination (*P* = 0.019), liver metastasis (*P* = 0.013) and TNM stage (*P* = 0.002). Kaplan-Meier analysis with log-rank test was performed to assess the prognostic significance of L1cam in gastric cancer. A significant difference of overall survival was found between patients with high L1cam expression and patients with low L1cam expression. Kaplan-Meier survival curves showed high L1cam expression was associated with poor overall survival (*P* < 0.001, Figure 2B-D, Table 1). Univariate analysis demonstrated patients with high L1cam expression tended to have a higher risk of death (HR = 2.73, 95% CI, 1.76-4.25; *P* < 0.001, Table 2). In addition, other parameters including tumor size, lymph node invasion and TNM stage were proved to be associated with overall survival as indicated by univariate analysis (Table 2). However, age, gender, differentiation status and therapeutic strategy had no prognostic significance in this studied population (Table 2, Additional file 1: Figure S1). Multivariate analysis showed only L1cam expression was an independent prognostic factors for gastric cancer patients (*P* = 0.022, Table 2).

**Table 1:** Correlations between L1cam expression and clinicopathological characteristics in gastric cancer patients

		**L1cam expression**		
**Characteristics**	**Total no.**	**Low** **No. cases (%)**	**High** **No. cases (%)**	***P *value**
Age				0.169
< 60	97	57(67)	40(56)	
≥ 60	59	28(33)	31(44)	
Gender				0.342
Male	106	55(64)	51(72)	
Female	50	30(36)	20(28)	
Tumor size				0.001^a^
<5 cm	101	65(76)	36(51)	
≥5 cm	55	20(24)	35(49)	
Differentiation status				0.290
Well	9	7(8)	2(3)	
Moderate	87	48(56)	39(55)	
Poor and others	60	30(36)	30(42)	
Lymph node invasion				0.007^a^
Absent	69	46(54)	23(32)	
Present	87	39(46)	48(68)	
Venous invasion				0.077
Absent	91	55(65)	36(51)	
Present	65	30(35)	35(49)	
Peritoneal dissemination				0.019^a^
Absent	123	73(86)	50(70)	
Present	33	12(14)	21(30)	
Liver metastasis				0.013^a^
Absent	114	69(81)	45(63)	
Present	42	16(19)	26(37)	
TNM^b^ stage				0.002^a^
I-II	48	35(41)	13(18)	
III-IV	108	50(59)	58(82)	
Therapeutic strategy				0.489
Surgery only	86	49(58)	37(52)	
Surgery + Chemotherapy	70	36(42)	34(48)	
Survival status				< 0.001^a^
Alive	72	52(61)	20(28)	
Dead	84	33(39)	51(72)	

^a^*P* **<** 0.05, Chi-square test.

^b^*TNM*, T, tumor; N, lymph node; M, distant metastasis.

**Table 2:** Univariate and multivariate analysis of various potential prognostic factors in gastric cancer patients

**Factors**	**Univariate analysis**				**Multivariate analysis**
	**Case NO.**	**HR**^**b**^**(95% CI**^**c**^**)**	***P***	**HR**^**b**^**(95% CI**^**c**^**)**	***P***
Age (<60/≥60)	87/69	0.94(0.61-1.44)	0.785	-	-
Gender (male/female)	106/50	1.17(0.74-1.84)	0.511	-	-
Differentiation (well, moderate/poor)	77/79	0.79(0.46-1.74)	0.381	-	-
Therapeutic strategy^d^(Sur/Sur + Chemo)	86/70	1.21(0.79-1.86)	0.377	-	-
Tumor size (≥5 cm/<5 cm)	105/51	2.27(1.54-3.37)	0.001^a^	1.73(0.99-3.03)	0.055
Lymph node invasion (present/absent)	111/45	1.55(1.25-1.92)	0.012^a^	1.05(0.73-1.53)	0.780
TNM stage (III-IV/I-II)	108/48	1.95(1.48-2.55)	0.001^a^	1.36(0.81-2.31)	0.249
L1cam expression (high/low)	71/85	2.73(1.76-4.25)	<0.001^a^	1.94(1.27-4.31)	0.022^a^

^a^*P* **<** 0.05.

^b^*HR*, hazard ratio.

^c^*CI*, confidence interval.

^d^*Sur*, Surgery only, Sur + Chemo: Surgery plus adjuvant Chemotherapy.

### L1cam promotes gastric cancer cell proliferation, migration and invasion *in vitro*

**Figure 3:**
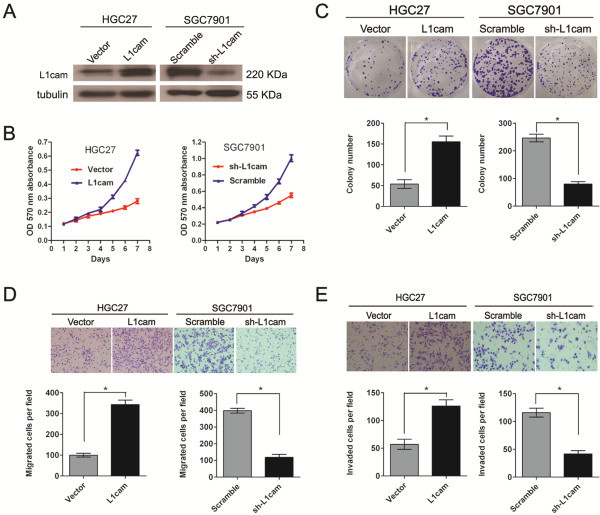
**L1cam promotes cell proliferation, migration and invasion in gastric cancer cell lines. (A)** L1cam protein level is increased after over-expression of L1cam in HGC27 cells and decreased after knockdown of L1cam in SGC7901 cells. **(B)** and **(C)** Ectopic expression of L1cam stimulates cell proliferation in HGC27 cells whereas knockdown of L1cam inhibits cell proliferation in SGC7901 cells as determined by MTT assays **(B)** and colony formation assays **(C). (D)** and **(E)** Ectopic expression of L1cam stimulates cell motility whereas knockdown of L1cam inhibits cell motility in gastric cancer cells as determined by migration assays **(D)** and invasion assays **(E)**. Bars represented mean ± SD of three independent tests, all *P < 0.05.

Based on the data listed above, we further evaluated the role of L1cam in cell proliferation, migration and invasion. The HGC27 cell line, which had relative low expression of L1cam, was transfected with a pcDNA3.1(+)-L1cam plasmid to overexpress L1cam. Another gastric cancer cell line SGC7901 that expressed relative high level of L1cam was treated with lentivirus that expressing short hairpin RNAs (shRNA) targeting L1cam to knockdown endogenous L1cam expression. The effect of ectopic expression and knockdown of L1cam in cells was confirmed by Western blot (Figure 3A). MTT assay showed that ectopic expression of L1cam could significantly promote the proliferative ability in HGC27 cells as compared with control cells (*P* < 0.05, Figure 3B). Similarly, colony formation capacity was increased after overexpression of L1cam (*P* < 0.05, Figure 3C). Conversely, knockdown of L1cam inhibited growth capacity in SGC7901 cells as indicated by the MTT and colony formation assays (*P* < 0.05, Figure 3B and C). Cell motility was measured by the migration and invasion assay. Compared with that of control cells, the migration and invasion ability were markedly stimulated in HGC27 cells that overexpressing L1cam (*P* < 0.05, Figure 3D and E). Likewise, Knockdown of L1cam could apparently repress the migration and invasion of SGC7901 cells (*P* < 0.05, Figure 3D and E).

### L1cam affects the responsiveness to oxaliplatin in gastric cancer cells

**Figure 4:**
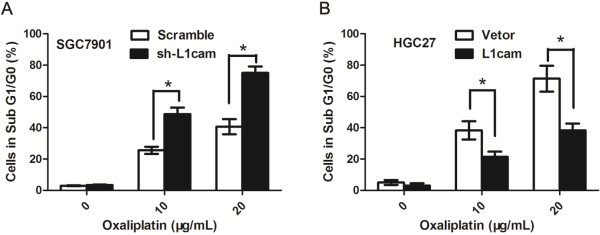
**L1cam affects the reponsiveness to oxaliplatin of gastric cancer cells. (A)** SGC7901 cells were transfected with sh-L1cam or sramble lentivirus. Cells were left untreated or administrated with different concentrations of oxaliplatin and cell cycle analyses were performed. Graph indicates the percentage of cells in sub G1/G0 phase (apoptotic cells), *P <0.05. **(B)** HGC27 cells were transfected with L1cam or negative control vectors. Cells were left untreated or administrated with different concentrations of oxaliplatin and cell cycle analyses were performed. Graph indicates the percentage of cells in sub G1/G0 phase (apoptotic cells), *P <0.05.

In order to determine if L1cam could affect apoptosis and the responsiveness to anti-cancer drugs in gastric cancer cells, firstly, we analyzed the effect of L1cam on the apoptosis of gastric cancer cells, the results showed that overexpression or knockdown of L1cam had no significant effect on the apoptosis rate in gastric cancer cells (Figure 4A and B); then cells were treated with different concentrations of oxaliplatin, the results showed that knockdown of L1cam could improve the responsiveness to oxaliplatin in SGC7901 cells while overexpression of L1cam could reduce the apoptosis rate in HGC27 cells (*P* < 0.05, Figure 4A and B).

### L1cam promotes tumorigenesis and metastasis of gastric cancer cells *in vivo*

To analyze the *in vivo* effects of L1cam on gastric cancer cells, we constructed two stable cell lines by using the lentivirus vector to mediate the knockdown of L1cam in SGC7901 cells; the resulting cells were designated as SGC7901/scramble and SGC7901/sh-L1cam cells respectively.

**Figure 5:**
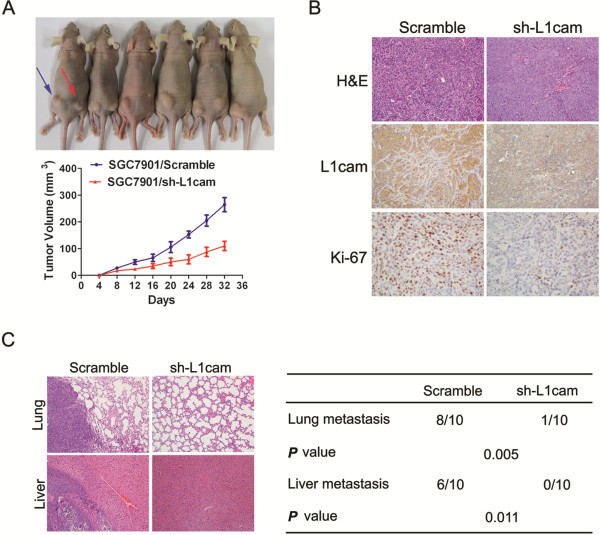
**L1cam promotes gastric cancer cell growth and metastasis *in vivo*. (A)** SGC7901/Scramble and SGC7901/sh-L1cam cells (1 × 10^6^cells/mouse) were injected subcutaneously into the left and right dorsal flanks of the nude mice (n = 6), tumor volumes were measured on the indicated days. Data points are presented as mean volume ± SD. **(B)** Histopathology of xenograft tumors, the tumor sections were under HE staining and IHC staining for L1cam and Ki-67. **(C)** SGC7901/Scramble and SGC7901/sh-L1cam cells (2 × 10^6^cells/mouse) were injected into the tail vein of two groups of nude mice (ten for each group). Six weeks post injection, the mice were killed and the lungs and livers were removed and paraffin embedded.

These two cell lines were injected into the left and right flanks of each nude mouse respectively. Tumor size was measured over time; after five weeks, mice were sacrificed and tumors were dissected out. The results showed that tumor growth was significantly inhibited in SGC7901/sh-L1cam cells as compared with that of SGC7901/scramble cells (*P* < 0.05, Figure 5A). In addition to the difference in tumor volume, we also found tumor tissues formed by injection of SGC7901/scramble cells displayed much stronger staining of L1cam and Ki-67, as detected by immunohistochemical analysis (Figure 5B). To explore the effect of L1cam on *in vivo* tumor metastasis, the two cell lines were injected into the tail vein of nude mice. Six weeks later, mice were sacrificed and lung and liver metastases were examined. Consistent with the *in vitro* results, the incidences of metastasis to lung and liver were significantly less in mice injected with SGC7901/sh-L1cam cells than those of SGC7901/scramble cells (*P* < 0.05, Figure 5C). These data suggest knockdown of L1cam could also inhibit the tumor growth and metastasis of gastric cancer cells *in vivo*.

### PI3K/Akt signal pathway is involved in L1cam mediated cellular activity

**Figure 6:**
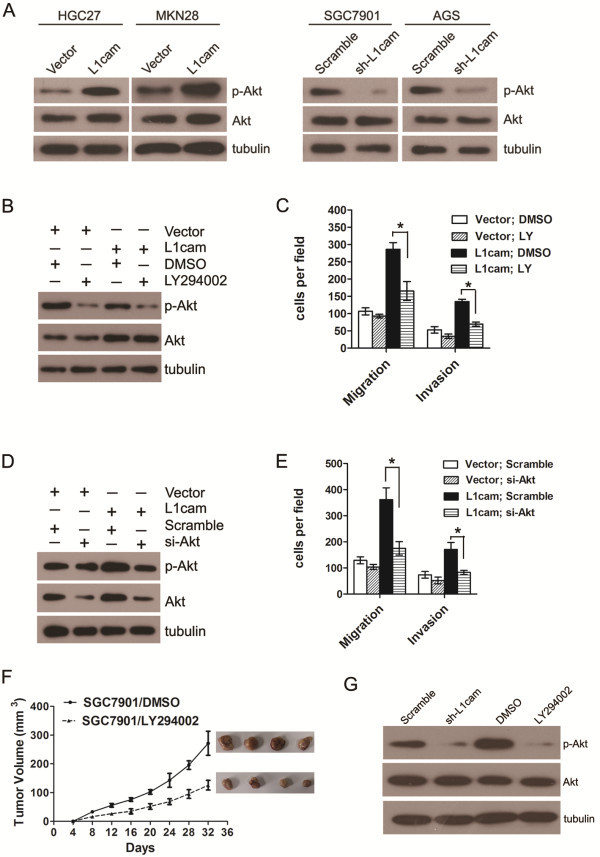
**PI3K/Akt signaling is involved in L1cam stimulated gastric cancer cell migration and invasion. (A)** Western blot analysis of whole- cell lysates using anti-phospho-Akt (Ser473) or anti-Akt antibody. **(B)** Western blot analysis in from HGC27 cells expressing L1cam or empty vector after pretreatment with 20 μM LY294002 for 30 min, using anti-phospho-Akt (Ser473) or anti-Akt antibody. **(C)** Migratory and invasive abilities of HGC27 cells expressing L1cam or the empty vector evaluated by Transwell assay after pretreatment with LY294002. **(D)** Western blot analysis of whole-cell lysates from HGC27 cells expressing L1cam or the empty vector 24 h after transfection with AKT siRNA or a scrambled siRNA, using anti-phospho-Akt (Ser473) or anti-Akt antibody. **(E)** Migratory and invasive abilities of HGC27 cells expressing L1cam or the empty vector as evaluated by Transwell assay after transfection with Akt siRNA or the scrambled siRNA. **(F)** SGC7901 cells were injected into the flank of nude mice, LY294002 (25 mg/kg) were intraperitoneally injected into the nude mice every four days, tumor volumes were measured at indicated days. **(G)** Tumors formed by different cells (SGC7901/Scramble or SGC7901/sh-L1cam, SGC7901/DMSO or SGC7901/LY294002) were collected and protein was extracted. Western blot analysis was conducted to detect the Akt and phospho-Akt level. Photomicrographs are at 100×. Bars correspond to mean ± SD of three independent experiments.

Relative high expression of L1cam was found in SGC7901 and AGS cells. Knockdown of L1cam dramatically decreased total phosphorylated Akt but not total Akt in SGC7901 and AGS cells (Figure 6A). Ectopic expression of L1cam in HGC27 and MKN28 cells significantly increased phospho-Akt and slightly increased total Akt levels (Figure 6A); this effect could be abolished upon treatment of the phosphoinositide-3-kinase inhibitor LY294002 (Figure 6B). In addition, the stimulation of cell migration and invasion caused by ectopic expression of L1cam could also be suppressed by LY294002 administration (Figure 6C). Similarly, the up-regulation of total Akt and phospho-Akt caused by ectopic expression of L1cam in HGC27 cells could be counteracted by knockdown of Akt using siRNA (Figure 6D); the effect on cellular motility caused by L1cam overexpression was also inhibited upon silencing of Akt (Figure 6E). Moreover, treatment of LY294002 in SGC7901 cells could significantly inhibit tumor growth in nude mice (Figure 6F), and the expression of phospho-Akt was reduced in tissues formed by cells that knockdown of L1cam or treated with LY294002 (Figure 6G). These results demonstrated that PI3K/Akt signaling was involved in L1cam stimulated cell growth and motility in gastric cancer cells.

## Discussion

In this study, we found that both L1cam mRNA and protein level was increased in gastric cancer cells and tissues. L1cam was detected in 73% of the tissues from gastric cancer patients by using IHC. Previously, Kodera et al. reported that L1cam was expressed in 21% of the specimens [27], but this study included p-T3 stage patients only, thus the inconsistency might be due to ethnic difference and difference in tumor stage. Moreover, expression of L1cam was significantly correlated with aggressive tumor characteristics (tumor size, lymph node invasion, peritoneal dissemination, liver metastasis and TNM stage) and poor prognosis; when the patients were subdivided into two groups according to tumor stage, we found L1cam could better distinguish patients with different outcomes in stage III-IV than in stage I-II patients, however, this might be due to the limited subjects in stage I-II. Multivariate analysis demonstrated that L1cam expression was an independent prognostic factor for gastric cancer patients. These observations suggested that overexpression of L1cam might be a common incidence in gastric cancer and could serve as an independent prognostic indicator to identify patients with different outcomes. In line with our study, up-regulation of L1cam was also found in other tumors, such as ovarian cancer, colorectal cancer, anaplastic thyroid carcinoma and intrahepatic cholangiocarcinoma [14,16,17,25]. However, further study is needed to confirm if L1cam could be used as a universal biomarker of prognosis for neoplasm.

As L1cam expression was associated with aggressive tumor phenotype in gastric cancer, we speculated that L1cam might play an important role in tumor biology. Indeed, knockdown of endogenous L1cam expression significantly inhibited cell proliferation, migration and invasion, whereas ectopic expression of L1cam enhanced these capacities. This is in line with previous studies that highlighted the role of L1cam in progression and metastasis of a variety of tumor types, such as uterine and ovarian carcinoma, human malignant melanoma, glioma and colorectal cancer [31-36]. It has been reported that L1cam could bind to a variety of integrins, form a protein-protein complex and activate several signaling pathways to promote cell adhesion and motility [37]; these L1cam/integrin-mediated signaling transduction may also integrate with growth factor signaling networks to stimulate cellular motility [37]. In addition, L1cam enables endocytosis of integrins by tumor cells, thus reducing cell adhesion to the extracellular matrix and promoting cell migration [38]. In the present study, in order to see whether L1cam can interact with integrins to promote cell motility in gastric cancer cells, SGC7901 cells as well as HGC27 cells overexpressing L1cam were treated with siRNAs against integrins (*β1*, *α5β1*, *α*v*β*3 and *α*v*β*5 integrins) that have been reported to be involved in L1cam mediated cellular activities. However, no significant effect on cellular proliferation and invasion was observed upon administration of these siRNAs. This is different from the results in some other tumors, for example, L1cam stimulated cell invasion by regulating FAK activation, possibly through interaction with integrin receptors after ADAM10 shedding in human glioma [39]; likewise, integrins are essential for L1cam-mediated NF-kappaB activation and cellular motility and invasiveness in pancreatic adenocarcinoma and breast cancer cells [40,41]. However, further study is needed to investigate the interaction of L1cam and integrins in gastric cancer cells.

Given that L1cam can promote gastric cancer cell proliferation, migration and invasion *in vitro*, we further investigated the *in vivo* effect of L1cam. To our interest, knockdown of L1cam by lentiviral-mediated short hairpin RNA (shRNA) interference significantly suppressed tumor growth and distant metastasis to lung and liver. This is in line with previous study that targeting L1cam decreased tumor growth and increased tumor-bearing survival in glioma and Cholangiocarcinoma [25,42]. Besides, L1cam monoclonal antibodies have been shown to reduce *in vivo* tumor growth of several types of cancer cells in mouse xenograft models, including ovarian cancer, colon carcinoma and intrahepatic Cholangiocarcinoma [25,43-45]. In this study, we also found that L1cam could affect the responsiveness to oxaliplatin in gastric cancer cells. In line with our results, it has been found that L1cam conferred anti-apoptotic protection and chemoresistance in pancreatic ductal adenocarcinoma cells [46]; moreover, a recent study demonstrated that inhibiting L1cam by using L1cam antibodies could increase the apoptotic response of tumor cells towards cytostatic drugs in pancreatic and ovarian carcinoma [47]. These results raise the possibility that L1cam could be used as a therapeutic target and L1cam antibodies might serve as chemosensitizers for malignant disease, including gastric cancer.

Recent studies have revealed that L1cam is involved in several signal pathways. For example, the Wnt/β-catenin/TCF pathway was found to induce the expression of L1cam in advanced colon cancer [36]. Ectopic expression of L1cam in ovarian carcinoma cells activates Erk and FAK signal pathways to promote cellular migration, invasion and apoptosis resistance [48,49]. In human glioma, L1cam stimulated cell motility via binding to integrin receptors, activating FAK, and increasing turnover of focal complexes [39]. L1cam could enhance cell proliferation by mainly activating ERK signaling in intrahepatic cholangiocarcinoma cells [50]. In the present study, we found ectopic expression in HGC27 cells activated PI3K/Akt signaling whereas knockdown of L1cam in SGC7901 cells inhibited Akt signaling. In addition, the increased cellular motilities promoted by L1cam could be eliminated by blocking of PI3K/Akt pathway in gastric cancer cells. Similar to our results, Min et al. reported Akt signaling is responsible for L1cam stimulation of intrahepatic cholangiocarcinoma progression [25]; Doberstein et al. found that L1cam could activate P13K/Akt pathway to induce cell proliferation and invasion in renal cell carcinoma [15]. These results suggest that the signaling pathways activated by L1cam may be tumor specific. However, further investigation is needed to explore the underlying molecular mechanism by which L1cam promotes gastric cancer progression and metastasis.

## Conclusions

In the present study, we found L1cam is overexpressed in gastric cancer cells and tissues. L1cam expression is associated with aggressive tumor phenotypes and poor survival in gastric cancer patients. Overexpression of L1cam promotes cell proliferation, migration and invasion, chemoresistanse as well as tumorigenesis and metastasis via activation of PI3K/Akt signaling pathway in gastric cancer. Therefore, L1cam expression level could be used for prediction of cancer progression, metastasis and prognosis of gastric cancer patients. Targeting L1cam might be a promising therapeutic strategy for gastric cancer patients.

## Materials and methods

### Human tissue specimens and cell lines

A cohort of 156 formalin-fixed, paraffin-embedded tissue samples collected from gastric cancer patients who underwent surgery in Sun Yat-sen University Cancer Center (Guangzhou, China) between 2004 and 2006 were retrieved. Fresh gastric cancer tissues and matched adjacent noncancerous tissues were obtained from 30 of the 156 patients and stored in liquid nitrogen until use. All the patients had a histological diagnosis of gastric cancer. A written informed consent was obtained from each patient involved in this study and the study protocol was approved by the ethics committee of Sun Yat-sen University Cancer Center. All the patients underwent total or subtotal gastrectomy, none of the patients received any treatment before surgery. Seventy patients who received adjuvant chemotherapy after surgery were on the 5-FU, platinum or taxol-based regimens. Each patient was followed-up regularly after operation at three-month interval. The median follow-up time was 30 months (range: 3 to 112 months). All the clinicopathological information including age, gender, tumor size, differentiation status, lymph node invasion, venous invasion, peritoneal dissemination, liver metastasis and TNM stage were retrieved from patients’ medical records.

Five human gastric cancer cell lines (MKN28, AGS, SGC7901, HGC27 and BGC823) were obtained from either the American Type Culture Collection or RIKEN Cell Bank; cells were cultured and stored according to providers’ instructions. Cells were routinely authenticated every six months (last examined in September 2012) by growth curve analysis, cell morphology monitoring and testing for mycoplasma.

### RNA isolation and real-time quantitative RT-PCR analysis

Total RNA was extracted from the tissues and cells with Trizol reagent (Invitrogen) according to the manufacturer’s instructions. The details for reverse transcription of RNA and real-time PCR are described previously [51]. L1cam mRNA expression were measured using a SYBR Premix Ex Taq™ kit (Takara); β-actin expression was used as a reference. The PCR primers for amplifications for L1cam and β-actin were:

L1cam forward: 5′-GACTACGAGATCCACTTGTTTAAGGA-3′;

L1cam reverse: 5′-CTCACAAAGCCGATGAACCA-3′;

β-actin forward: 5′-TGGATCAGCAAGCAGGAGTA-3′;

β-actin reverse: 5′-TCGGCCACATTGTGAACTTT-3′.

Real-time PCR was carried out with an ABI PRISM® 7500 Seqtence Detection System. The relative level of L1cam mRNA was normalized to that of β-actin and calculated by the 2^-△△ct^ method.

### Western blot analysis

Western blot analysis was performed according to a standard method as described previously [52]. For immunoblotting of L1cam, a mouse L1cam antibody (sc-33686) was purchased from Santa Cruz Biotechnology. For detection of Akt and p-Akt, rabbit antibodies against total Akt and Ser^473^ phosphorylated Akt were obtained from Cell Signaling Technology. A mouse monoclonal α-tubulin antibody (1:20000; Abcam) was used as loading control.

### Immunohistochemistry (IHC) analysis

The paraffin-embedded tissue blocks were cut into 4 μm slides. A mouse L1cam antibody (sc-33686) was used for immunostaining. IHC analysis of L1cam was performed according to a previously described method [53]. To quantify L1cam protein expression, both the intensity and extent of immunoreactivity were evaluated and scored. In the present study, IHC intensity was scored as follows: 0, negative staining; 1, weak staining; 2, moderate staining; 3, strong staining. The scores of the extent of immunoreactivity ranged from 0 to 3 and were according to the percentage of cells that had positive staining in each microscopic field of view (0, <25%; 1, 25%-50%; 2, 50%-75%; 3, 75%-100%). A final score ranging from 0 to 9 was achieved by multiplying the scores for intensity and extent. L1cam expression level was considered high when the final scores were ≥ 4 and low when the final scores were < 4.

### Vector construction and transfection, lentivirus production and transduction

To overexpress L1cam, the coding sequence of L1cam was amplified and subcloned into the pcDNA3.1 (+) vector (Invitrogen, CA, USA) according to the manufacturer’ instructions. HGC27 cells were then transfected with a negative control vector or L1cam expressing plasmid using lipofectamine 2000 (Invitrogen). The resultant cells were named HGC27/Vector and HGC27/L1cam cells, respectively. To generate L1cam stable knockdown cells, lentivirus containing L1cam short hairpin RNA (shRNA) or scrambled oligonucleotides were obtained from GenePharma Biotech (Shanghai, China). An annealed short interfering RNA (siRNA) for L1cam selected from 3 different target sequences was inserted into the LV-3 (pGLVH1/GFP + Puro) vector. SGC7901 cells were transduced with lentivirus and stable cell lines were selected per the manufacturer’s instructions. The targets for L1cam shRNA were, for sh-L1cam#1, 5′-GGAAATGAGACCACCAATA-3′; for sh-L1cam#2, 5′-CAACAGTGCTTCAGGACGA-3′; for sh-L1cam#3, 5′-CGATGAAAGATGAGACCTT-3′. In this study, we used sh-L1cam#1 because it could effectively knockdown endogenous L1cam in gastric cancer cell lines based on our preliminary experiments. The target sequence for scrambled shRNA was 5′-GTCTCCACGCGCAGTACATTT-3′. The cell lines stably expressing L1cam shRNA or scrambled oligonucleotides were designated as SGC7901/sh-L1cam and SGC7901/scramble cells, respectively.

### Cell proliferation assays

The 3-(4, 5-dimethylthiazole-2-yl)-2, 5-biphenyl tetrazolium bromide (MTT) assay was performed to test cell viability and proliferation. The spectrophotometric absorbance at 570 nm was measured for each sample, all the experiments were repeated 3 times in triplicate and the mean was calculated.

For the colony formation assay, 500 cells were placed in a six-well plate and cultured for 14 days with RPMI 1640 medium (GIBCO) containing 10% FBS. Colonies were fixed with methanol and stained with 0.1% crystal violet (1 mg/ml).

### *In vitro* invasion and migration assay

The cell invasive and migratory potential was evaluated using transwell chambers (8 μm pore; BD Biosciences). For the invasion assay, 1 × 10^5^ cells suspended in 100 μl serum-free medium were added to the upper chamber of the inserts, which were coated with a mitrigel mix; fetal bovine serum (500 μl) was added to the lower chamber as a chemoattractant. After incubation for 24 hours, non-invading cells on the upper surface were wiped off with a cotton swab and cells that invaded to the lower side of the membrane were fixed with methanol, stained with 0.1% crystal violet, air dried and photographed. For the migration assay, tumor cells (5 × 10^4^ cells in 100 μl serum-free medium) were placed in the top chamber of each insert without matrix gel, and 500 μl fetal bovine serum was added to the lower compartment. 16 hours later, the cells on the upper side were removed, and the cells that migrated to the lower chamber were fixed and stained with crystal violet. The number of invading or migrating cells was determined by microscopically counting five different fields.

### Cell cycle analysis

Cells were seeded in six-well plates and cultured for 12 hours, and then cells were left untreated or treated with different concentrations of oxaliplatin (10 μg/mL or 20 μg/mL) for 24 hours. Afterward, cells were collected and washed with phosphate-buffered saline, cell cycle analysis was carried out as previously described [54].

### *In vivo* proliferation and metastasis assays

Female BABL/c athymic nude mice (four to five weeks old) were obtained from the Animal Center of Guangdong province (Guangzhou, China). All the animal experiments were performed according to the National Institutes of Health animal use guidelines on the use of experimental animals.

To evaluate the *in vivo* proliferative effect of L1cam, the SGC7901/Scramble and SGC7901/sh-L1cam cells (1 × 10^6^ cells/mouse) were injected subcutaneously into the left and right dorsal flanks of the nude mice. Tumor size was measured every four days and tumor volume was estimated. After five weeks, the mice were sacrificed and the tumors were dissected out. Tumor tissues were fixed with 10% formalin and embedded in paraffin. Representative tumor sections were obtained from paraffin-embedded tumor tissue and stained with haematoxylin-eosin (H&E) or specific antibodies.

To investigate the effect of L1cam on tumor metastasis, the SGC7901/Scramble and SGC7901/sh-L1cam cells (2 × 10^6^ cells/mouse) were injected into the tail vein of two groups of nude mice (ten for each cell group). Six weeks post injection, the mice were sacrificed and the lungs and livers were removed and paraffin embedded. Consecutive sections (4 μm) were made and stained with haematoxylin-eosin. The micro-metastases in the lungs and livers were examined and counted under a dissecting microscope as described previously [55].

### Blocking of PI3K/Akt pathway and assays

LY294002, a specific inhibitor of PI3K, was purchased from Cell Signaling Technology. For administration of LY294002, tumor cells were incubated with 50 μM LY294002 (Cell Signaling Technology) for one hour before performing *in vitro* assays. Small-interfering RNA (siRNA) targeting Akt and a scrambled siRNA were purchased from Ribobio (Guangzhou, China). The target sequence for AKT siRNA is 5′-GCACCTTCATTGGCTACAA-3′, and the target for scrambled siRNA is 5′-CGTACGCGGAATACTTCGA-3′. For siRNA transfection, cells were plated in a six-well plate the day before transfection. Twenty-four hours later, cells were transfected with 50 nM siRNAs using lipofectamine 2000 (Invitrogen) according to the manufacturer’s instructions. The efficiency of gene silencing was confirmed by immunoblotting. Migration and invasion assays were performed twenty-four hours after siRNA transfection in gastric cancer cells. To evaluate the *in vivo* effect of LY294002, SGC7901 cells were subcutaneously implanted into the flank of nude mice, Seven days later, LY294002 (25 mg/kg) were intraperitoneally injected into the nude mice every four days. The tumor volume was measured every four days. The mice were sacrificed after 5 weeks and the tumors were dissected out.

### Statistical analysis

Statistical analysis was performed using the SPSS software package (version 16.0, SPSS Inc). Statistical significance was tested by a Student’s t-test or a Chi-square test as appropriate. Survival analysis was performed using the Kaplan-Meier method, and the log-rank test was used to compare the differences between patient groups. Parameters with a *P* value < 0.05 by univariate analysis were subject to multivariate analysis using the Cox proportional hazards model to identify independent prognostic factors for gastric cancer patients. All differences were statistically significant with a value of *P* < 0.05.

## Abbreviations

IHC: Immunohistochemistry; TNM: Tumor, lymph node, distant metastasis; MTT: 3-(4, 5-dimethylthiazole-2-yl)-2, 5-biphenyl tetrazolium bromide; siRNA: Small interfering RNA.

## Competing interests

The authors declare that they have no competing interests.

## Authors’ contributions

CDL conceived of the study, carried out the Western blotting analysis, IHC analysis, molecular studies and drafted the manuscript. ZZL performed the animal experiments. YJ and RC collected the clinical data. WDS and WWJ performed the statistical analysis. XRH participated in the design of the study and helped to draft the manuscript. All authors read and approved the final manuscript.

## Supplementary Material

Additional file 1: Figure S1 Kaplan-Meier analysis of overall survival based on Therapeutic strategies in 156 gastric cancer patients. Click here for file
