# Supplementary material for: L1cam promotes tumor progression and metastasis and is an independent unfavorable prognostic factor in gastric cancer
Source: J Hematol Oncol. 2013 Jun 27;6:43. doi: 10.1186/1756-8722-6-43 (PMC3717076; doi:10.1186/1756-8722-6-43)
Supplement: Additional file 1: Figure S1 — Kaplan-Meier analysis of overall survival based on Therapeutic strategies in 156 gastric cancer patients. [file 1756-8722-6-43-S1.ppt]

## Slide 1
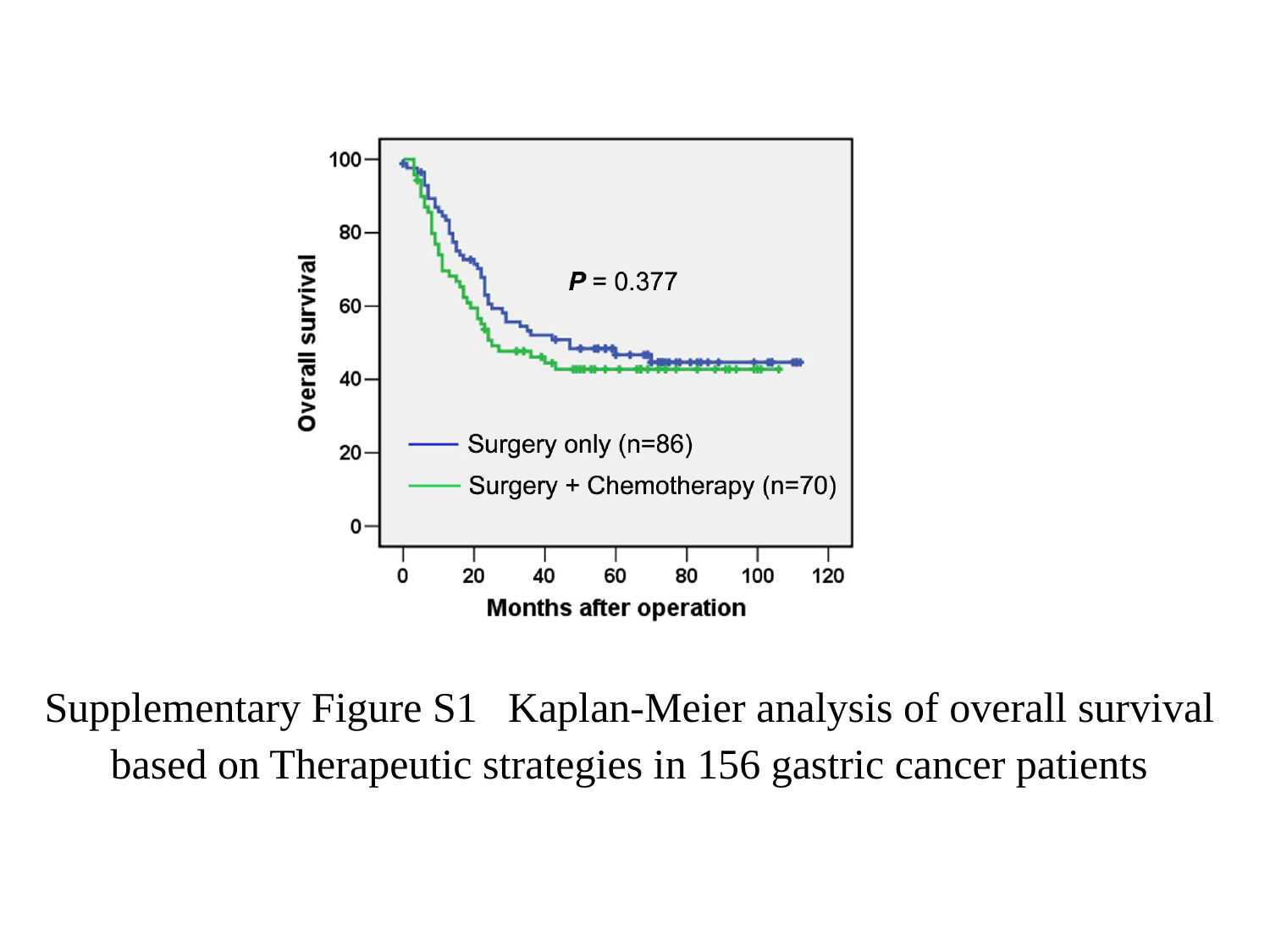

# Supplementary Figure S1 Kaplan-Meier analysis of overall survival based on Therapeutic strategies in 156 gastric cancer patients
